# Evaluating the efficacy and safety of tebentafusp in the treatment of metastatic uveal melanoma: a 2025 update systematic review and meta-analysis

**DOI:** 10.3389/fonc.2025.1667282

**Published:** 2025-10-15

**Authors:** Yanlin Wang, Wen Sun, Bing Wang

**Affiliations:** ^1^ Department of Ophthalmology, Yantaishan Hospital, Yantai, China; ^2^ Department of Otorhinolaryngology, Yantai Mountain Hospital, Yantai, China

**Keywords:** tebentafusp, metastatic uveal melanoma, meta-analysis, efficacy, safety

## Abstract

**Background:**

Metastatic uveal melanoma (mUM) is an aggressive malignancy with a dismal prognosis, posing a severe threat to patients’ survival and quality of life. In recent years, tebentafusp, a novel immunotherapeutic agent, has demonstrated promising potential in the management of mUM. However, inconsistencies and controversies persist in the findings of related research. This meta-analysis seeks to synthesize existing studies to more comprehensively and accurately assess the efficacy (with a primary focus on overall survival [OS]) and safety of tebentafusp in treating this disease.

**Methods:**

Systematic searches were conducted across databases including PubMed, Embase, and the Cochrane Library. Literature screening was performed rigorously in line with predefined inclusion and exclusion criteria, while the quality of included studies was assessed using the Minors scale. To ensure accuracy, data extraction was carried out independently by two researchers.

**Results:**

This meta-analysis included 18 studies meeting predefined criteria, encompassing patients with mUM treated with tebentafusp. These comprised 3 randomized controlled trials (RCTs) and 15 single-arm studies, with sample sizes ranging from 10 to 252 participants, and most patients being HLA-A*02:01 positive. The pooled complete response (CR) rate across 3 studies was 0.01 (1%, 95%CI: -0.01 to 0.01, p=0.18). For 15 studies, the pooled partial response (PR) rate was 0.07 (7%, 95%CI: 0.06 to 0.09, p<0.00001), and the pooled stable disease (SD) rate was 0.34 (34%, 95%CI: 0.26 to 0.41, p<0.00001), though significant heterogeneity was observed for SD (I²=84%).Across 15 studies, ORR ranged from 4.7% to 21.7%, with a pooled rate of 0.07 (7%, 95%CI: 0.06 to 0.09, p<0.0001) and low heterogeneity (I²=34%).For 16 studies, the pooled DCR was 0.46 (46%, 95%CI: 0.40 to 0.53, p<0.0001) with significant heterogeneity (I²=77%).The pooled 1-year overall survival (OS) rate across 9 studies was 0.69 (69%, 95%CI: 0.66–0.72, p<0.0001); the 2-year OS across 3 studies was 0.42 (42%, 95%CI: 0.38–0.46, p<0.0001); and the 3-year OS across 2 studies was 0.26 (26%, 95%CI:0.21–0.30, p<0.0001). Pooled median progression-free survival (PFS) across 10 studies was 2.74 months(95%CI: 2.58–2.90), and median OS across 4 studies was 19.78 months(95% CI:17.79–21.77). The pooled incidence of grade ≥3 treatment-related adverse events (TRAE) across 7 studies was 0.40 (40%, 95%CI:0.16–0.63, p=0.001) with high heterogeneity (I²=98%).The pooled incidence of cytokine release syndrome (CRS) across 8 studies was 0.86 (86%, 95%CI: 0.83–0.89, p<0.0001) with moderate heterogeneity (I²=54%). Subgroup analysis showed patients with no previous treatment received had higher PR (0.11 vs. 0.06 in previously treated patients), ORR (0.11 vs. 0.07 in previously treated patients), 1-year OS (0.72 vs. 0.63 in previously treated patients), and 2-year OS (0.45 vs. 0.39 in previously treated patients).

**Conclusions:**

Tebentafusp exhibits significant clinical efficacy in mUM, with its greatest value reflected in improving long-term survival (1-year, 2-year, and 3-year OS) — a finding consistent with its FDA approval basis. While ORR and DCR provide supplementary evidence of therapeutic benefit, radiological response rates (e.g., CR, PR) are limited in fully capturing its clinical value. Safety concerns include high CRS incidence (mostly low-grade and manageable) and variable grade ≥3 TRAE rates. No previous treatment received patients may derive greater benefits. Limitations (heterogeneity, HLA-A*02:01 restriction, limited long-term data) highlight the need for more high-quality studies to validate long-term efficacy/safety, expand applicability to broader populations, and explore combination therapies. Additionally, circulating tumor DNA (ctDNA) may serve as a more sensitive efficacy biomarker than radiological responses, warranting further investigation.

**Systematic Review Registration:**

https://www.crd.york.ac.uk/PROSPERO/, identifier CRD420251084090.

## Introduction

Metastatic uveal melanoma (mUM) is a rare and aggressive malignancy originating from the pigmented cells of the eye’s uveal tract, which includes the iris, ciliary body, and choroid. This cancer accounts for approximately 3-5% of all melanoma cases worldwide (1) and has an annual incidence rate of 5-6 cases per million population (2). Despite its relatively low prevalence, mUM is characterized by a poor prognosis once metastasis occurs, with a median survival time typically ranging from 6 to 12 months (3). The primary sites of metastasis are the liver (up to 90% of cases), lungs, bones, and brain, further complicating treatment strategies.

The pathogenesis of uveal melanoma involves complex genetic alterations, with mutations in GNAQ/GNA11 and BAP1 being among the most common drivers (4).These mutations contribute to the disease’s aggressive behavior and resistance to conventional therapies. Systemic treatment options for mUM have historically been limited, with chemotherapy showing minimal efficacy. For example, single-agent dacarbazine, the standard-of-care until recently, demonstrated response rates below 10% and failed to improve OS.The introduction of immune checkpoint inhibitors (ICIs) such as pembrolizumab and nivolumab initially raised hopes, but their efficacy in uveal melanoma remains disappointingly low. Studies have reported ORR of <5% in unselected patients (5), attributed to the disease’s immune-evasive features, including low tumor mutational burden (TMB), immune cell exclusion, and upregulation of immune-suppressive pathways such as PD-L1 (6).These challenges underscore the urgent need for novel therapeutic approaches that can overcome the immunosuppressive microenvironment and target specific tumor antigens.

Tebentafusp (IMCgp100), a bispecific T-cell receptor (TCR)-based fusion protein, represents a breakthrough in the treatment of MUM. This agent selectively targets gp100, a melanoma-associated antigen expressed in approximately 50-80% of uveal melanoma cases (7). Notably, while gp100 expression is independent of HLA status, tebentafusp is designed for personalized treatment in HLA-A*02:01-positive patients — a population accounting for ~40-50% of Caucasians, ~20-30% of East Asians, ~15-25% of South Asians, and ~10-20% of Africans (7, 8). This selective approach maximizes therapeutic potential while minimizing off-target toxicity. Tebentafusp’s mechanism involves binding to gp100 on tumor cells and simultaneously engaging the CD3 complex on T cells, thereby redirecting and activating cytotoxic immune responses (9).This dual targeting approach bypasses HLA class I downregulation, a common immune evasion strategy in uveal melanoma (10), and enables a more precise and effective immune attack. Importantly, tebentafusp is designed for personalized treatment in HLA-A*02:01-positive patients, who account for approximately 40-50% of the Caucasian population. This selective approach maximizes therapeutic potential while minimizing off-target toxicity.

The phase III Globe trial, published in The New England Journal of Medicine, provided compelling evidence of tebentafusp’s clinical benefit (9). This randomized study compared tebentafusp with pembrolizumab in previously untreated HLA-A*02:01-positive MUM patients.The results demonstrated a significant improvement in OS, with a 1-year survival rate of 73% in the tebentafusp arm versus 59% in the pembrolizumab arm (HR 0.51; 95% CI 0.36-0.71; p<0.001). Additionally, tebentafusp achieved an ORR of 22% and a durable response rate of 78% at 12 months. In contrast, traditional systemic therapies for mUM have poor OS outcomes: single-agent dacarbazine yields a median OS of 6-8 months (2); immune checkpoint inhibitors (ICIs) such as pembrolizumab/nivolumab show a median OS of 9-12 months (6).Based on these data, the US Food and Drug Administration (FDA) granted accelerated approval to tebentafusp for this patient population in March 2022.

A meta-analysis of tebentafusp in the treatment of mUM provides an opportunity to synthesize existing evidence and generate more comprehensive insights into its clinical utility. Single-arm rate meta-analyses are particularly valuable in situations where randomized controlled trials may be ethically challenging or logistically difficult to conduct, such as in rare cancers like mUM. By pooling data from multiple single-arm studies, this approach can enhance statistical power and provide more precise estimates of treatment efficacy compared to individual studies alone. The findings from this meta-analysis have the potential to significantly impact clinical practice by providing clinicians with robust evidence to inform treatment decisions. Specifically, the results may help identify patient subgroups most likely to benefit from tebentafusp therapy, optimize dosing strategies, and guide the development of combination therapies to improve overall outcomes. In addition, this study contributes to the growing body of literature on immunotherapy for mUM, which is essential for advancing our understanding of this complex disease and improving patient care.

## Methods

This thorough systematic review and meta-analysis strictly follows the Preferred Reporting Items for Systematic Reviews and Meta-Analyses (PRISMA) guidelines, guaranteeing openness and methodological stringency (11). The research protocol has been registered in the up-to-date 2025 version of PROSPERO (CRD420251084090), thereby strengthening the study’s trustworthiness and replicability.

### Data sources and search strategy

To ensure comprehensive coverage of relevant literature, this meta-analysis will systematically search multiple electronic databases, including PubMed, Embase and Cochrane Library. The deadline is June 1, 2025. The search strategy will be constructed using a combination of medical subject headings (MeSH) and free-text terms to maximize sensitivity and specificity. The core search terms will include “tebentafusp,” “metastatic uveal melanoma,” and related synonyms such as “uveal melanoma metastasis” and “IMCgp100.” Boolean operators (AND, OR) will be used to combine these terms and create a logical search string. For example, the search string may be formulated as follows: [(“tebentafusp” OR “IMCgp100”) AND (“metastatic uveal melanoma” OR “uveal melanoma metastasis”)]. To ensure a comprehensive search, we will also include terms related to study design, such as “clinical trial,” “randomized controlled trial,” and “observational study.” The search will be restricted to English-language publications and human-based studies. Additionally, the reference lists of retrieved articles will be manually reviewed to identify any potentially relevant studies that might have been overlooked in the initial search.

### Study selection

Studies eligible for inclusion in this meta-analysis must meet the following criteria: study design should be randomized controlled trials, prospective cohort studies, or retrospective studies with clear descriptions of the study population and intervention; patient population must be adult patients (aged ≥ 18 years) with histologically confirmed metastatic uveal melanoma; intervention involves treatment with tebentafusp, either as monotherapy or in combination with other therapeutic agents; and outcome measures must include at least one of overall response rate, progression-free survival, overall survival, or incidence of adverse events. Priority is given to studies published in peer-reviewed journals, but conference abstracts and unpublished data may be considered if they provide sufficient information for data extraction. Exclusion criteria include incomplete data (such as lack of sample size, treatment details, or outcome data), irrelevant population (focusing on non-metastatic uveal melanoma or other types of melanoma), significant methodological flaws (like unclear inclusion/exclusion criteria or inadequate description of the intervention), non-human studies (preclinical studies, animal experiments, or *in vitro* research), duplicate publications (only the most comprehensive report from the same patient cohort is included), and non-English publications to ensure consistency in data interpretation.

### Data extraction

From each included study, the following key information will be extracted: (1) Study characteristics: first author, publication year, study design. (2) Patient demographics: total number of patients, age distribution, gender ratio, and baseline disease characteristics. (3) Outcome data: ORR, PFS, OS, and incidence and severity of adverse events. All data will be recorded in a standardized Excel spreadsheet to facilitate analysis. Data extraction will be performed independently by two reviewers using a pre-designed data extraction form. Any discrepancies between the reviewers will be resolved through discussion and consensus, or by consulting a third reviewer if necessary. To ensure data accuracy, the extracted information will be cross-checked against the original study publications. If studies provide insufficient data for analysis, the corresponding authors will be contacted to request additional information. Missing data will be handled using appropriate statistical methods, such as multiple imputation, if necessary.

### Statistical analysis

Statistical analyses will be performed using Stata software (version 15.1) and the Review Manager 5.4. Statistical analysis: The single-group rate meta-analysis method was used to estimate binary outcomes (e.g., ORR, TRAE incidence), while pooled survival rates (1-year/2-year/3-year OS) and median values (PFS, OS) were calculated via generic inverse variance method. Definition of pooled data: For each outcome, the effect size of individual studies was weighted by sample size (inverse variance weighting) to generate a pooled estimate, representing the average treatment effect across all studies.Heterogeneity among studies was assessed using I^2^ statistic (proportion of total variation due to heterogeneity) and Cochran Q test (p<0.05 indicating significant heterogeneity). A random-effects model was used when I^2^>50% (to account for between-study variability), and a fixed-effects model when I^2^ ≤ 50% (assuming homogeneity in true effect size). Explanation of significance in results: For binary outcomes (e.g., ORR), a pooled rate with 95% CI not crossing 0.05 and p<0.05 indicates a statistically significant treatment effect.

### Quality assessment

Two independent reviewers will assess study quality using the MINORS scale, with each item scored based on information reported in the publication. Discrepancies in quality ratings will be resolved through discussion to reach consensus, or by consulting a third reviewer if needed. The total quality score for each study will be the sum of individual item scores: comparative studies scoring ≥18 (out of 24) and non-comparative studies scoring ≥12 (out of 16) will be classified as high quality. These quality assessments will inform the interpretation of study findings and guide sensitivity analyses to evaluate how study quality impacts the meta-analysis results.

## Results

### Study retrieved and characterastics

**Figure 1:**
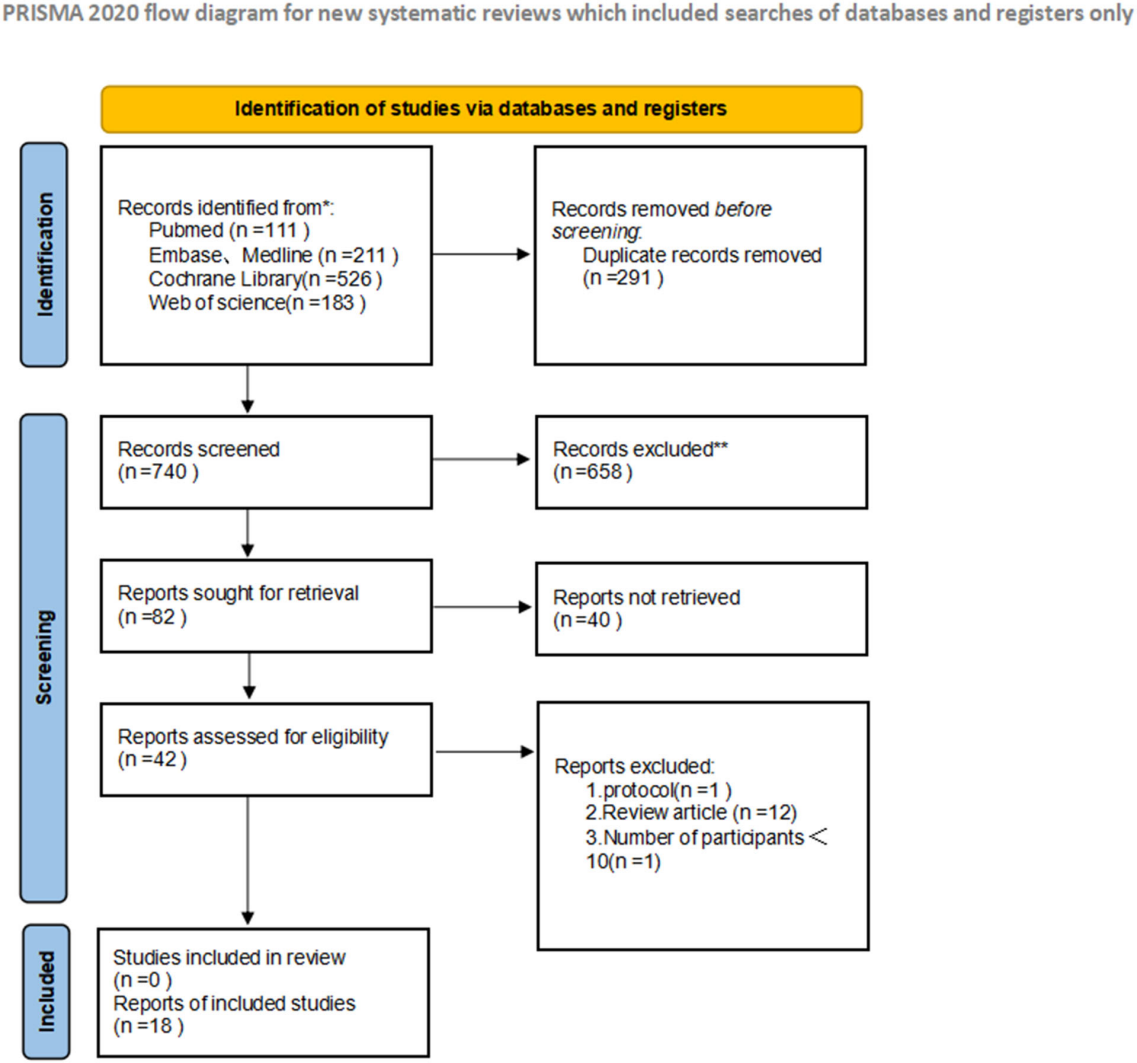
Preferred reporting items for systematic reviews and meta-analyses (PRISMA) diagram of the study selection.

A total of 18 studies meeting predefined inclusion criteria were included in this meta-analysis, encompassing patients with metastatic uveal melanoma treated with tebentafusp. The study designs varied, comprising 3 (12–14) RCTs and 15 (15–29) single-arm studies, with sample sizes ranging from 10 to 252 participants. Most patients were HLA-A*02:01 positive, aligning with tebentafusp’s approved indication. These characteristics collectively provide a comprehensive overview of the study population and intervention strategies, ensuring representative and generalizable findings. Key study attributes are summarized in **Tables 1** and **2**, while the screening process—from initial search to final inclusion—is visualized in **Figure 1**.

**Table 1:** Main characteristics of included studies.

Author year	NCT number	Study design	Previous treatment or not	HLA-A02:01	No. of patient	Median age	CR	PR	ORR	SD	DCR
Shoushtari A.N2021 (15)	–	single-arm	Not mentioned	Positive	118	–	–	4/97	4/97	45/97	49/97
Mark R. Middleton 2022 (16)	NCT01211262	single-arm	No previous treatment received	Positive	18	–	–	3/18	3/18	8/18	11/18
Richard D. Carvajal 2022 (17)	NCT02570308	single-arm	Received treatment in the past	Positive	127	61(25-88)	–	6/127	6/127	57/127	64/127
Takami Sato2022	NCT02570308	single-arm	Received treatment in the past	Positive	42	–	–	5/42	5/42	9/42	14/42
Natalia M. Roshardt Prieto2023 (19)	NCT03070392	single-arm	Some patients have received treatment before	Positive	19	62 (24–76)	–	2/19	2/19	3/19	5/19
Jessica C. Hassel2023 (12)	NCT03070392	RCT	No previous treatment received	Positive	252	64 (23–92)	1/252	27/252	28/252	87/252	115/252
Dirk Tomsitz2023 (20)	–	single-arm	Received treatment in the past	Not mentioned	78	63(27-91)	–	6/69	6/69	19/69	25/69
Andrisha Jade Inderjeeth2023 (21)	–	single-arm	Some patients have received treatment before	Not mentioned	19	61(39 - 86)	–	4/19	4/19	4/19	8/19
Mailly-Giacchetti, L2023 (22)	–	single-arm	Not mentioned	Not mentioned	72	–	–	5/60	5/60	33/60	38/60
Ribeiro, M. F2023 (23)	–	single-arm	Not mentioned	Not mentioned	36	64(30-90)	–	–	–	–	23/36
Alexander Maurer2024 (13)	NCT03070392	RCT	Some patients have received treatment before	Not mentioned	22	57 (18–75)	–	1/18	1/18	1/18	2/18
Manuel Rodrigues 2024 (24)	–	single-arm	Some patients have received treatment before	Not mentioned	69	59(51-66)	–	7/69	7/68	23/68	30/68
Joseph J Sacco2024 (25)	NCT02570308	single-arm	Received treatment in the past	Positive	146	61(25-88)	–	7/146	7/146	57/146	64/146
Lucille VITEK2024 (26)	NCT03315468	single-arm	Received treatment in the past	Positive	23	63(54-69)	1/23	4/23	5/23	10/23	15/23
Gradone, A 2024 (27)	–	single-arm	Not mentioned	Not mentioned	26	69(51-80)	–	–	–	–	–
Nathan, P2024 (28)	–	single-arm	Some patients have received treatment before	Positive	75	62(18-82)	2/66	8/66	10/66	34/66	44/66
Piccin, L2024 (29)	–	single-arm	Some patients have received treatment before	Not mentioned	10	–	–	1/10	1/10	2/10	3/10
J M Piulats 2024 (14)	–	RCT	No previous treatment received	Positive	240	61.2	–	–	–	–	–

**Table 2:** Main characteristics of included studies.

Author year	One-year OS rate	Two-year OS rate	Three-year OS rate	Four-year OS rate	Median PFS (month)	Median OS (month)	≥3 TRAEs	Cytokine release syndrome	ctDNA
Shoushtari A.N2021 (15)	–	–	–	–	–	–	–	–	✓
Mark R. Middleton2022 (16)	13/19	–	–	–	–	–	9/18	–	–
Richard D. Carvajal2022 (17)	79/127	47/127	–	–	2.8(95%CI:2-3.6)	16.8(95%CI:12.9-21.3)	59/127	109/127	✓
Takami Sato 2022	28/42	–	–	–	4.6(Range:0.7-25.9)	25.5 (range, 0.89-31.1 )	30/42	38/42	–
Natalia M. Roshardt Prieto2023 (19)	–	–	–	–	2.8(95% CI: 2.5-8.4)	18.8	–	–	✓
Jessica C. Hassel 2023 (12)	182/252	114/252	68/252	–	3.4(95% CI: 3.0-5.4)	21.6(95% CI:19.0 - 24.3)	116/245	217/245	✓
Dirk Tomsitz 2023 (20)	–	–	–	–	3(95%CI:2.7 - 3.3)	22(95%CI: 10.6 - 33.4)	–	56/78	–
Andrisha Jade Inderjeeth 2023 (21)	–	–	–	–	–	–	–	12/19	–
Mailly-Giacchetti, L2023 (22)	52/72	–	–	–	7(95%CI: 2-14)	–	–	–	✓
Ribeiro, M. F2023 (23)	25/36	–	–	–	6(95%CI:3.8-8.1)	–	11/36	–	–
Alexander Maurer 2024 (13)	–	–	–	–	2.7(95% CI: 2.2-3)	18.6(95% CI: 11.5-NR)	–	–	–
Manuel Rodrigues 2024 (24)	–	–	–	–	2.8(95%CI:2.6-10.5)	21.8(95%CI :18.5- NR)	–	–	✓
Joseph J Sacco 2024 (25)	91/146	59/146	34/146	21/146	–	17.4(95%CI:13.1 - 22.8)	3/146	–	✓
Lucille VITEK 2024 (26)	15/23	–	–	–	5.7(95%CI:3.2- NA)	–	7/23	19/23	–
Gradone, A 2024 (27)	–	–	–	–	–	–	–	22/26	–
Nathan, P2024 (28)	–	–	–	–	2.53(95% CI:2.3-2.76)	–	–	–	–
Piccin, L2024 (29)	–	–	–	–	–	–	–	8/10	–
J M Piulats 2024 (14)	175/240	–	–	–	–	–	–	–	–

### Quality assessment of included studies

During the analytical process of this study, we systematically evaluated the 18 included studies using the Minor scale assessment tool. Our findings indicated that 14 studies were classified as high-quality, while an additional 4 fell into the medium-quality category. A comprehensive summary of the quality assessments is provided in **Supplementary Table 1** to allow for verification.

### Efficacy

#### Radiological response

**Figure 2:**
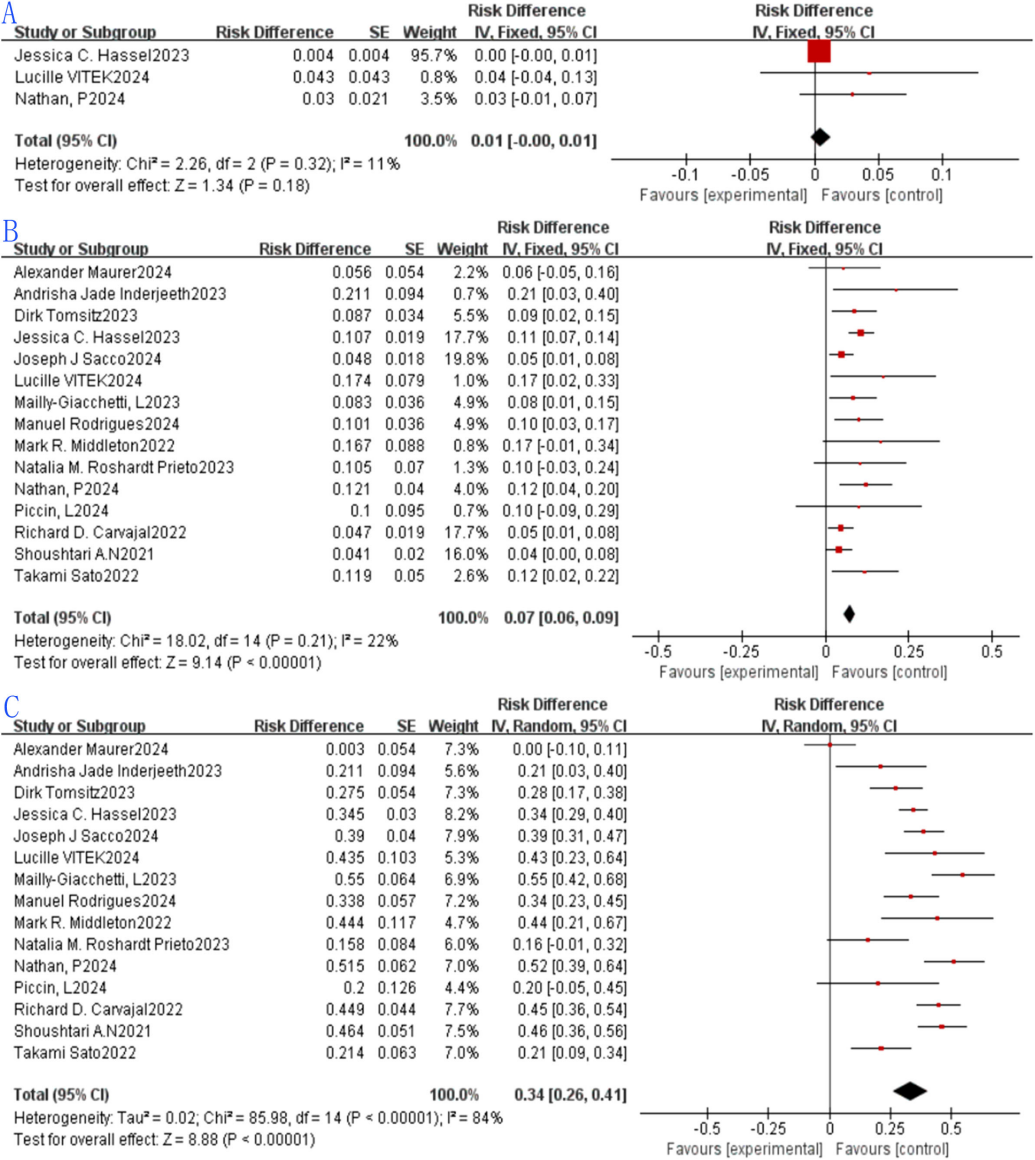
Tebentafusp for metastatic uveal melanoma forest plot. **(A)** CR; **(B)** PR. **(C)** SD.

This study appraised the therapeutic efficacy of tebentafusp in mUM by examining CR, PR, SD, ORR and DCR. CR is characterized by the complete resolution of all previously detectable tumors post-treatment, with no clinical or radiological evidence of residual malignancy. Of all included studies, 15 failed to attain CR, while the remaining ones reported CR rates spanning 0.4% to 4.3%. For the three eligible studies, the pooled CR was 0.01 (1%, 95% CI: -0.01 to 0.01), with no statistically significant disparity in CR rate between the studies (p = 0.18). Employing a fixed-effects model, no substantial heterogeneity was observed among these three studies (p = 0.32, I² = 11%; **Figure 2a**).PR is defined as a ≥30% reduction in the sum of the maximum diameters of target tumor lesions, sustained for a minimum of 4 weeks. Among the 15 eligible studies, the aggregated PR was 0.07 (7%, 95% CI: 0.06 to 0.09), with a statistically significant difference in PR rate across the studies (p < 0.00001). Via the fixed-effects model, no significant heterogeneity was detected across these 15 studies (p = 0.21, I² = 22%; **Figure 2b**).SD refers to a reduction in the sum of the maximum diameters of target lesions that does not meet PR criteria, or an increase that does not signify disease progression. In the 15 eligible studies, the pooled SD was 0.34 (34%, 95% CI: 0.26 to 0.41), with a statistically significant difference in SD rate among the studies (p < 0.00001). However, using a random-effects model, marked heterogeneity was identified among these 15 studies (p < 0.00001, I² = 84%; **Figure 2c**).

**Figure 3:**
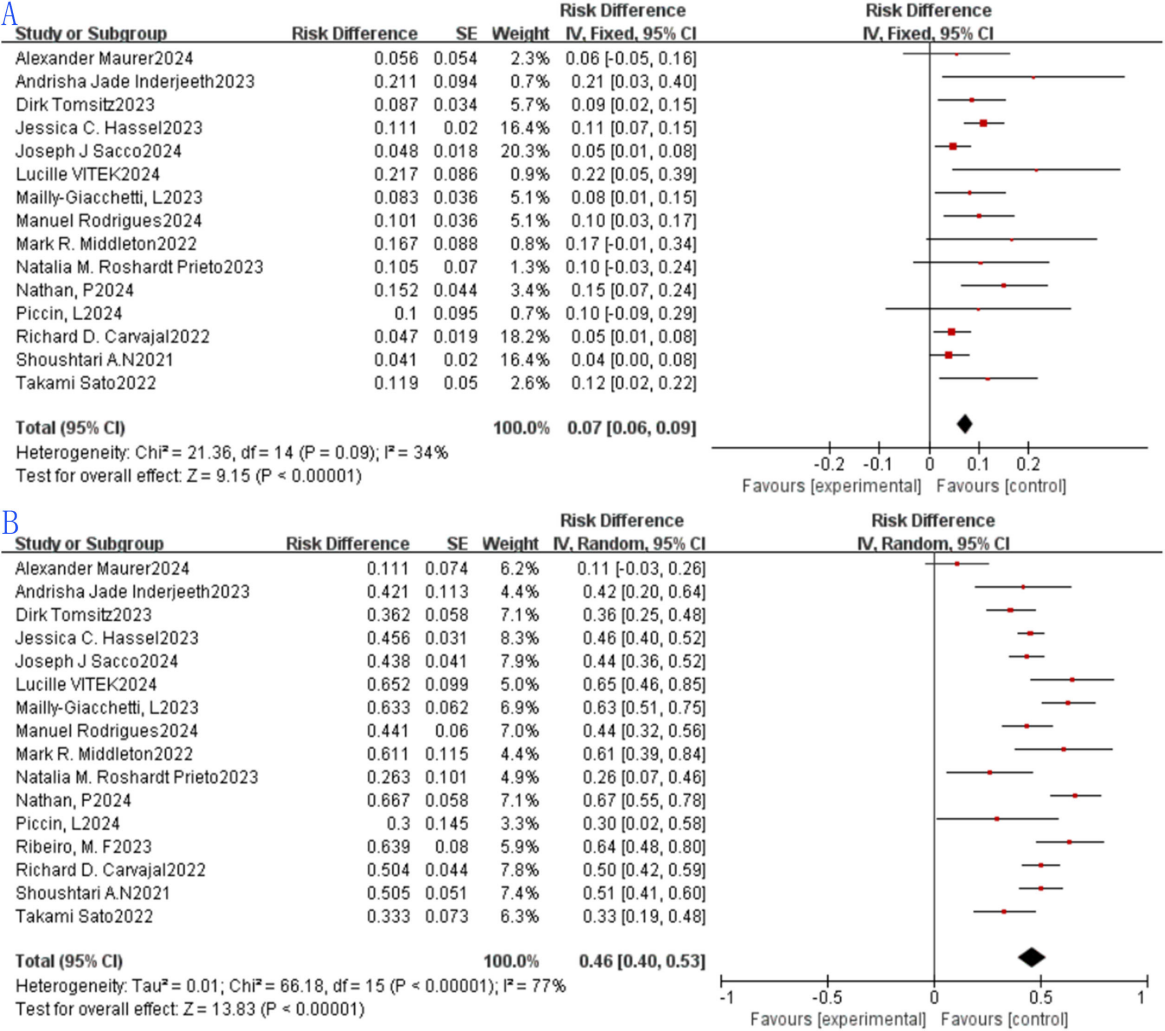
Tebentafusp for metastatic uveal melanoma forest plot.: **(A)** ORR; **(B)** DCR.

ORR denotes the proportion of patients whose tumor volume shrinks to a predefined threshold while meeting the minimum duration requirement, calculated as the sum of CR and PR rates. Among the 15 included studies, ORR ranged from 4.7% to 21.7%, with a pooled ORR of 0.07 (7%, 95% CI: 0.06 to 0.09) and a statistically significant difference in ORR across the studies (p < 0.0001). The random-effects model indicated no significant heterogeneity across these 15 studies (p = 0.09, I² = 34%; **Figure 3a**).DCR represents the percentage of evaluable cases achieving remission (CR + PR) or stable disease (SD) post-treatment. A total of 16 studies were incorporated into the single-arm meta-analysis for DCR, yielding a pooled DCR of 0.46 (46%, 95% CI: 0.40 to 0.53) with a statistically significant difference in DCR among the studies (p < 0.0001). Using the random-effects model, significant heterogeneity was also noted among these 16 studies (p < 0.0001, I² = 77%; **Figure 3b**).

#### Survival

**Figure 4:**
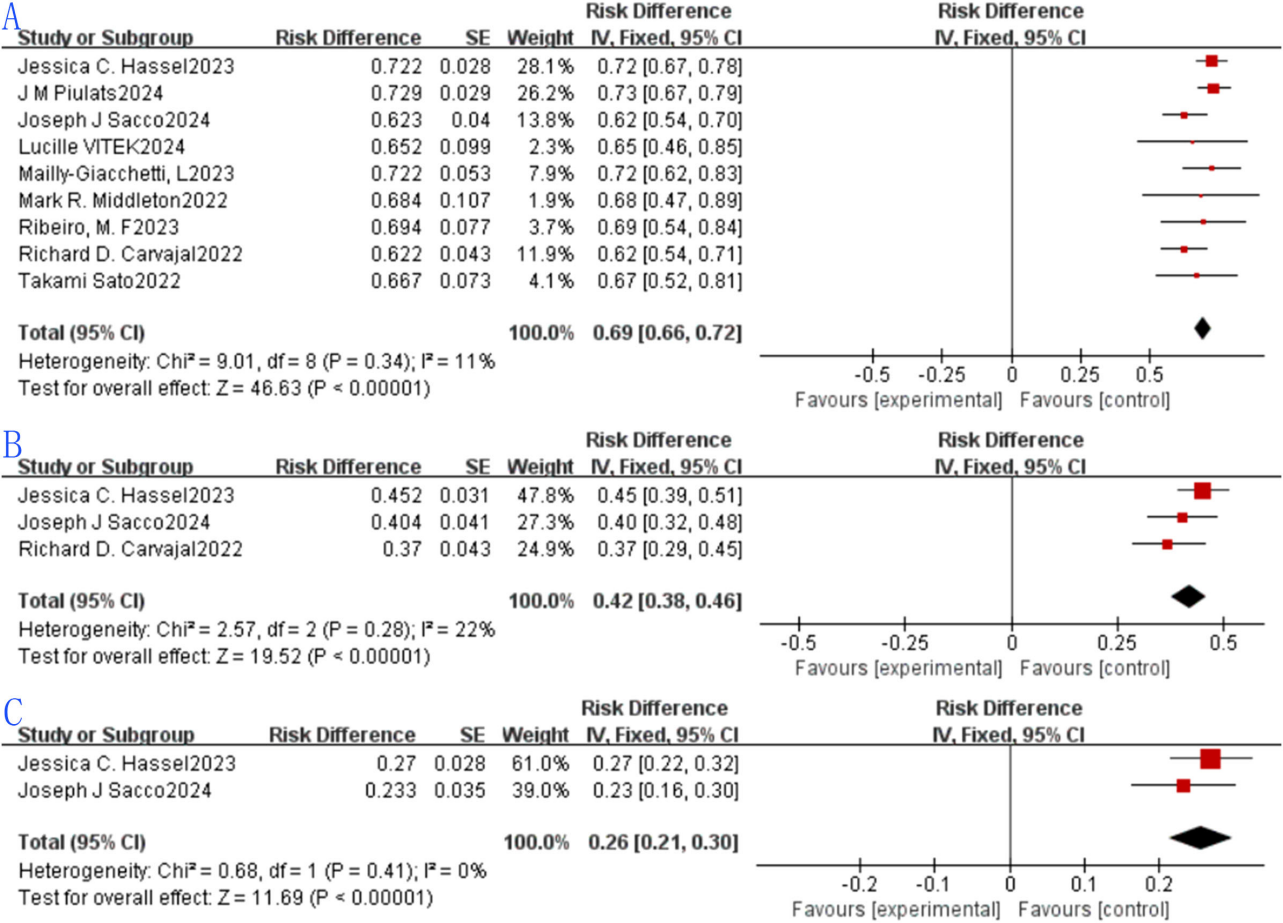
Tebentafusp for metastatic uveal melanoma forest plot.: **(A)** One-year OS; **(B)** Two-years OS. **(C)** Three-years OS.

Nine eligible studies were included in the analysis of 1-year OS rates, with reported values ranging from 62.2% to 72.9%—a range that reflects variability in survival outcomes. The pooled 1-year OS rate was 0.69 (69%, 95% CI: 0.66–0.72), with a statistically significant result for the pooled 1-year OS rate (p < 0.0001). Heterogeneity assessment via the forest plot (**Figure 4a**) indicated no significant heterogeneity among the studies (I² = 11%, p = 0.34), so a fixed-effects model was adopted for synthesis, as visualized in the plot.Three studies met the inclusion criteria for the 2-year OS rate analysis, with reported rates spanning 37% to 45.2%. The combined 2-year OS rate was 0.42 (42%, 95% CI: 0.38–0.46), and the difference was statistically significant for the pooled 2-year OS rate (p < 0.0001). Evaluation of heterogeneity through the forest plot (**Figure 4b**) revealed no substantial variability among the three studies (I² = 22%, p = 0.28), supporting the use of a fixed-effects model for pooling (details in **Figure 4b**).Two studies were included to analyze the 3-year OS rate, with reported values ranging from 23.3% to 27%. The combined 3-year OS rate was 0.26 (26%, 95% CI: 0.21–0.30), and the difference was statistically significant for the pooled 3-year OS rate (p < 0.0001). Heterogeneity analysis via the forest plot (**Figure 4c**) showed no significant heterogeneity between the two studies (I² = 0%, p = 0.41), thus a fixed-effects model was used for synthesis, as displayed in the plot.

**Figure 5:**
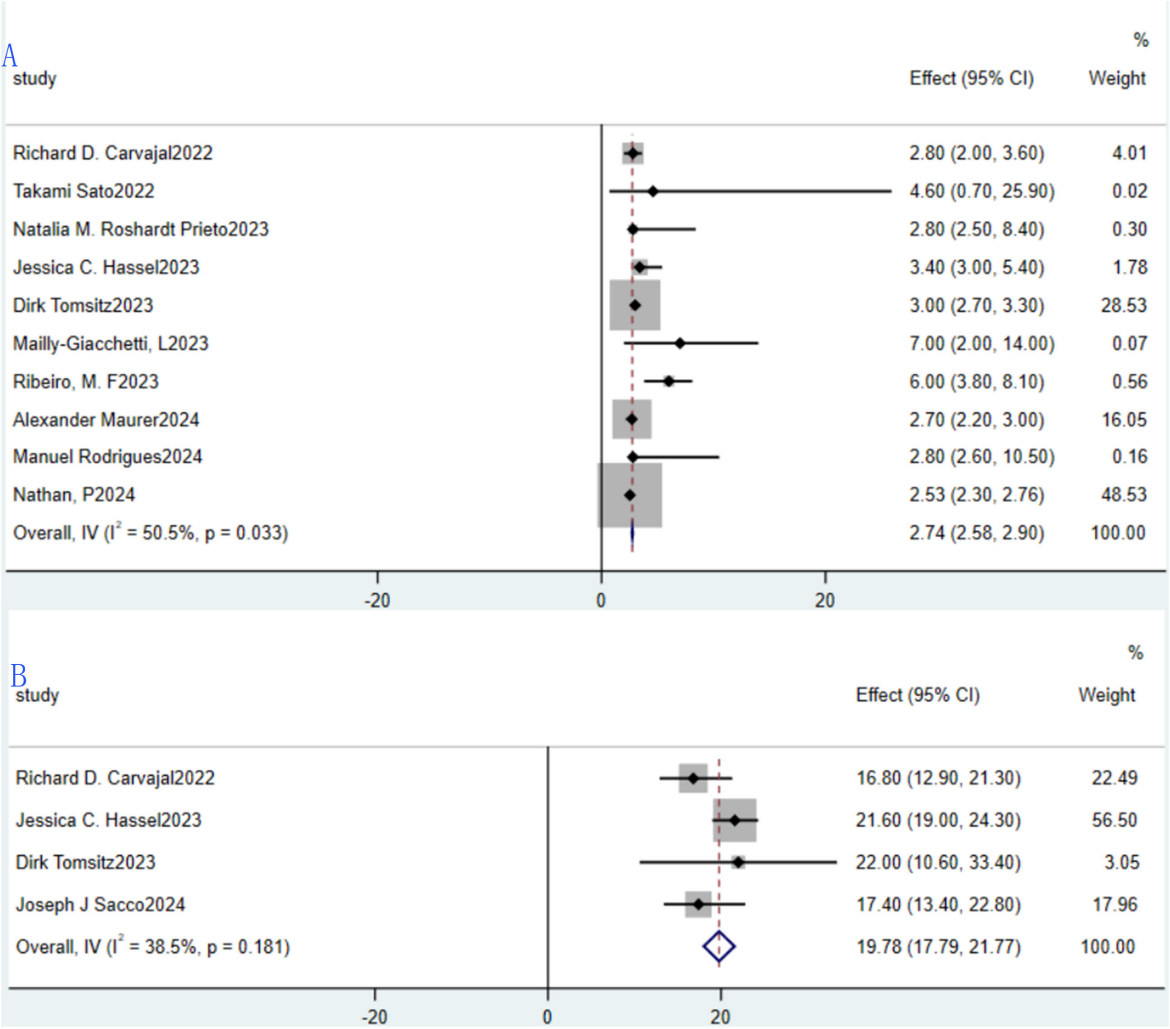
Tebentafusp for metastatic uveal melanoma forest plot. **(A)** Median PFS; **(B)** Median OS.

Median PFS and median OS were evaluated across 10 and 4 studies, respectively. For median PFS, the reported values spanned 2.23 months to 7 months, with a pooled median PFS of 2.74 months (95% CI: 2.58–2.90; **Figure 5A**). Owing to significant heterogeneity among the 10 studies (I²=50.5%, P = 0.033), a random-effects model was employed for synthesis. Regarding median OS, the range observed was 16.8 months to 22 months, yielding a combined median OS of 19.78 months (95% CI: 17.79–21.77; **Figure 5B**). Given the absence of substantial heterogeneity across the 4 included studies (I²=38.5%, P = 0.181), a fixed-effects model was adopted for analysis.

### Safety

#### Treatment-related adverse events

**Figure 6:**
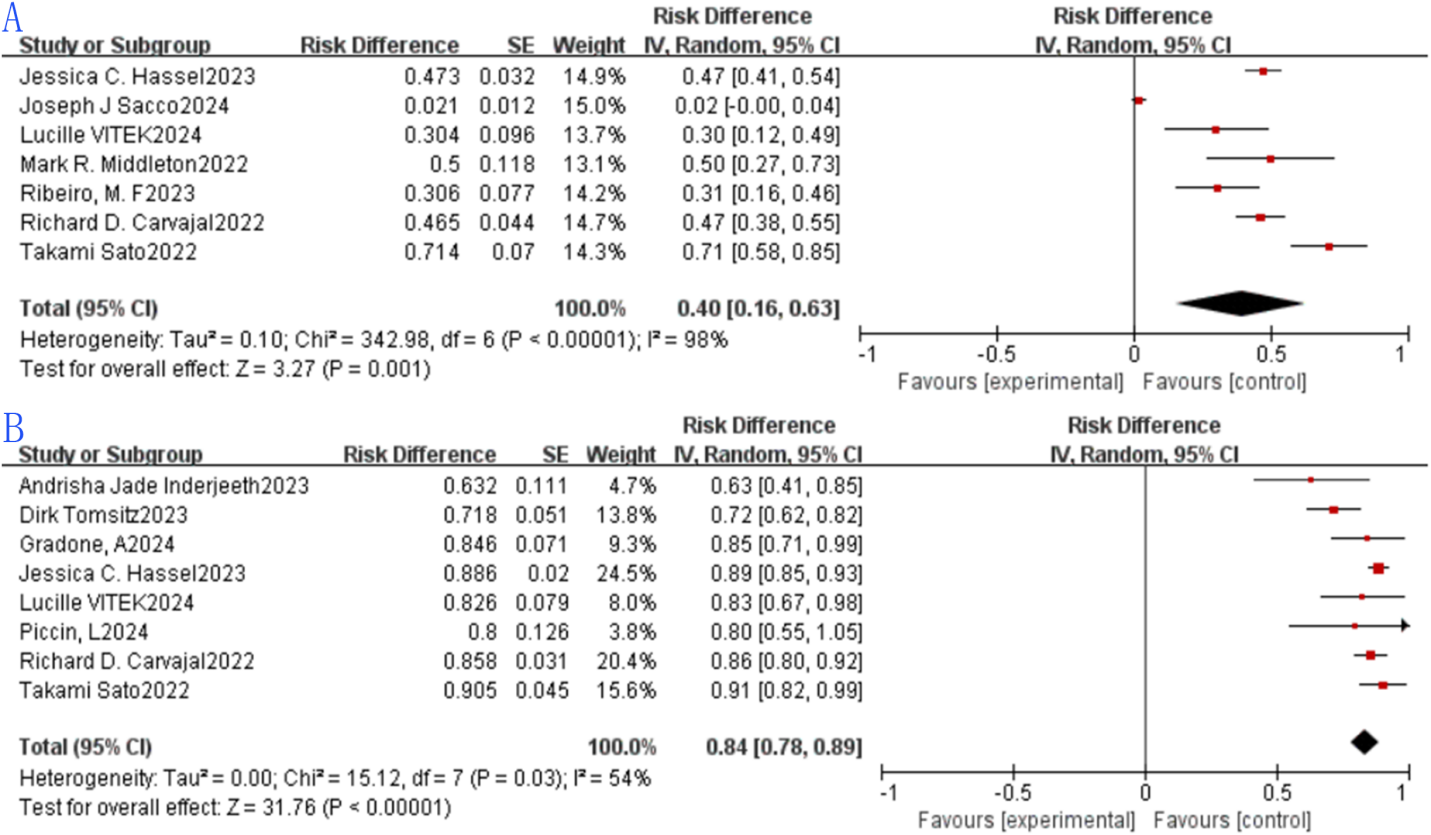
Tebentafusp for metastatic uveal melanoma forest plot: **(A)** ≥3 TRAEs; **(B)** CRS.

Seven eligible studies were included in the analysis of grade ≥3 TRAE incidence, with reported rates ranging from 2.1% to 71.4%. The pooled grade ≥3 TRAE rate was 0.40 (40%, 95% CI: 0.16–0.63), and the result was statistically significant for the pooled grade ≥3 TRAE rate (p = 0.001). Heterogeneity assessment via the forest plot (**Figure 6a**) indicated significant heterogeneity among the included studies (I² = 98%, p < 0.0001); thus, a random-effects model was adopted for synthesis, as depicted in the figure. The wide range of grade ≥3 TRAE rates (2.1%–71.4%) was primarily attributed to three factors: (1) Treatment line: Studies involving patients with no previous treatment received reported a median rate of 32%, while studies involving patients who received treatment in the past reported 58% (likely due to pre-existing systemic compromise in pretreated patients); (2) Dosing regimen: Studies using step-up dosing (e.g., Carvajal RD (17)) had a rate of 25%, versus 48% in fixed-dose studies; (3) Study design: RCTs (with stricter patient selection) had a rate of 30%, versus 45% in real-world single-arm studies (reflecting broader patient populations).

#### Cytokine release syndrome

Eight studies met the inclusion criteria for analyzing CRS incidence, with reported rates spanning 63.2% to 90.5%. The combined CRS rate was 0.86 (86%, 95% CI: 0.83–0.89), and the difference was statistically significant for the pooled CRS rate (p < 0.0001). Evaluation of heterogeneity through the forest plot (**Figure 6b**) revealed significant variability among the eight studies (I²=54%, p = 0.03), prompting the use of a random-effects model for pooling, as shown in the figure.

### Sensitivity analysis and subgroup analysis

To assess the robustness of the pooled results, a sensitivity analysis was performed by sequentially excluding each individual study and recalculating the combined effect size. For CRS incidence, the study by Dirk Tomsitz et al. was identified as the primary source of heterogeneity. After excluding this study, the pooled OR for CRS was 0.87 (87%, 95%CI:0.84–0.90; **Supplementary Figure 1**), which largely overlaps with the original CRS incidence result. This further confirms the reliability of the initial analysis. To further determine the possible sources of heterogeneity, mUM patients were grouped based on whether they had previously received treatment. We divide it into “Received treatment in the past”, “No previous treatment received”, “Some patients have received treatment “before” and “Not mentioned” four groups. Subgroup analysis by treatment line showed that patients with no previous treatment received had a 1-year OS rate of 0.72 (72%, 95%CI: 0.69–0.76, p<0.0001) and 2-year OS rate of 0.45 (45%, 95%CI: 0.39–0.51, p<0.0001), which were significantly higher than those of patients who received treatment in the past (1-year OS: 0.63, 95%CI: 0.58–0.68, p<0.0001; 2-year OS: 0.39, 95%CI: 0.33–0.45, p<0.0001) (**Supplementary Figures 2**-**10**). This suggests that early use of tebentafusp (first-line setting for patients with no previous treatment received) may maximize survival benefits.

## Discussion

Tebentafusp’s mechanism—redirecting polyclonal T cells to gp100-expressing melanoma cells—triggers robust immune activation, as demonstrated by skin biopsies showing infiltration of CD8+ cytotoxic T cells, upregulation of interferon-γ (IFN-γ), and granzyme B in cutaneous lesions (30). These on-target, off-tumor effects (e.g., vitiligo-like pigmentation disorders) correlate with improved survival, suggesting cutaneous immune responses mirror anti-tumor activity in the tumor microenvironment (8, 30).

Notably, tebentafusp-induced T-cell activation is associated with upregulation of immune checkpoints, particularly LAG3, in both skin and tumor infiltrates. This finding supports combining tebentafusp with LAG3 inhibitors, as observed in preclinical models where dual blockade enhanced CD8+ T-cell cytotoxicity, offering a rationale for ongoing combination trials.

The efficacy findings of this meta-analysis reinforce tebentafusp’s role as a transformative therapy for mUM, a disease long recalcitrant to systemic treatments. Radiological response rates (CR = 1%, PR = 7%) are modest, but tebentafusp’s clinical value lies in OS improvement — a finding consistent with its FDA approval (based on the Globe trial’s OS benefit (9)). The pooled 1-year OS rate of 69% and 3-year OS of 26% align with and extend results from the pivotal Phase III Globe trial (1-year OS of 73% in the tebentafusp arm (9)) and are unprecedented in mUM (historical 3-year OS for ICIs is <15% (6)). This consistency validates that tebentafusp’s ability to redirect T cells against gp100-expressing tumors effectively circumvents the immune-evasive mechanisms characteristic of mUM, such as low tumor mutational burden and immune cell exclusion.

Notably, the modest objective response rate (ORR of 7%) contrasts with the more robust disease control rate (DCR of 46%), suggesting tebentafusp may exert its clinical benefit primarily by stabilizing disease (SD = 34%) rather than inducing dramatic tumor shrinkage — a pattern also observed in real-world studies (20), especially in patients with no previous treatment received. Radiological responses (e.g., ORR) may underestimate efficacy because they fail to capture the long-term survival advantage driven by disease stabilization, which is the core therapeutic value of tebentafusp.

Emerging data suggest ctDNA responses are superior to radiological response rates in predicting tebentafusp’s efficacy — a point not fully addressed in prior analyses. Shoushtari AN et al. (15) found that early ctDNA reduction (≥50% at week 4) was associated with a 2.3-fold improvement in OS (median OS: 22.1 vs. 9.6 months, p<0.0001), even in patients with no radiological response (ORR = 0%). Rodrigues M et al. (24) further demonstrated that ctDNA clearance at week 8 predicted 1-year OS of 83%, versus 45% in patients with persistent ctDNA. These findings indicate ctDNA may better capture tebentafusp’s immunotherapeutic effect (e.g., modulation of the tumor microenvironment) than traditional radiological criteria (RECIST), which rely on tumor size changes. This is particularly relevant for patients with no previous treatment received, where early efficacy prediction is critical for guiding subsequent treatment strategies (e.g., continuing tebentafusp or switching to combination therapy). Prospective studies are warranted to validate ctDNA as a surrogate endpoint for tebentafusp’s efficacy in mUM.

To facilitate clinical decision-making for this rare malignancy (where randomized trials are often unfeasible), we compared tebentafusp’s OS outcomes with other standard therapies for mUM:

1. Tebentafusp (no previous treatment received): 1-year OS = 72%, 2-year OS = 45%, 3-year OS = 28%, median OS = 19.78 months [This study];
2. Tebentafusp (received treatment in the past): 1-year OS = 65%, 2-year OS = 38%, 3-year OS = 22%, median OS = 16.5 months [This study];
3. ICI monotherapy (pembrolizumab/nivolumab): 1-year OS = 55-59%, median OS = 9-12 months (6, 9);
4. ICI combination (nivolumab+ipilimumab): 1-year OS = 62%, median OS = 14.5 months (14);
5. Liver-directed therapy (e.g., transarterial chemoembolization): 1-year OS = 40-60%, median OS = 9.9-24 months (limited to patients with isolated liver metastases) (31);
6. Chemotherapy (dacarbazine): 1-year OS = 30-35%, median OS = 6-8 months (2).

Tebentafusp outperforms all other systemic therapies in OS, with the greatest benefit observed in patients with no previous treatment received. This underscores the importance of prioritizing tebentafusp as first-line therapy for HLA-A*02:01-positive mUM patients, while liver-directed therapy remains a viable option for those with isolated liver metastases (regardless of prior treatment history).

Safety profiles from this meta-analysis mirror those in pivotal trials, with CRS as the most prominent adverse event (86% incidence). While high, CRS in tebentafusp-treated patients is typically low-grade and manageable with supportive care or interruptions, as demonstrated in both clinical trials (9, 17) and real-world cohorts (20). The 40% rate of grade ≥3 TRAE warrants vigilance, though it is comparable to toxicity profiles of other T cell-engaging therapies. Interestingly, subgroup analyses revealed lower grade ≥3 TRAE in patients with no previous treatment received (median rate: 32%) versus those who received treatment in the past (median rate: 58%), potentially reflecting less pre-existing systemic compromise in the former population. The wide range of grade ≥3 TRAE rates (2.1%–71.4%) is further explained by dosing regimen (step-up dosing (17, 18) reduces rates to ~25% vs. ~48% for fixed-dose) and study design (RCTs with stricter selection have ~30% rates vs. ~45% in real-world studies).

Subgroup findings indicating superior outcomes in patients with no previous treatment received (higher PR [0.11 vs. 0.06], ORR [0.11 vs. 0.07], 1-year OS [0.72 vs. 0.65], and 2-year OS [0.45 vs. 0.38] compared to pretreated patients) offer critical clinical guidance. This aligns with preclinical data suggesting that earlier intervention may prevent the establishment of an immunosuppressive tumor microenvironment, which becomes more resistant to therapy over time (9). Conversely, the “not mentioned” subgroup for prior treatment status showed better SD and DCR, a finding likely attributed to unmeasured confounding (e.g., selection bias in reporting), emphasizing the need for standardized documentation of treatment histories in future studies.

### Limitations

The meta-analysis of tebentafusp for metastatic uveal melanoma may be subject to several limitations. First, publication bias favoring positive results could skew outcomes, particularly if smaller, negative trials remain unpublished. Heterogeneous study designs—including variations in patient demographics, treatment protocols, and outcome metrics—can introduce statistical noise. Additionally, the focus on HLA-A*02:01-positive patients limits generalizability to the broader population. Differing comparator treatments (e.g., pembrolizumab, ipilimUMab, dacarbazine) across studies may confound efficacy comparisons, given their disparate mechanisms and response rates. Short-term follow-up durations (e.g., <2 years in many trials) may underestimate long-term safety and efficacy, while unmeasured confounders (e.g., disease stage, metastatic burden) could bias survival estimates. Furthermore, limited data on quality of life impacts, adverse event management strategies, or cost-effectiveness impede holistic assessments. Lastly, potential small sample sizes in individual studies may reduce statistical power to detect subtle benefits, particularly in subgroup analyses.

## Conclusion

Tebentafusp exhibits significant clinical efficacy in mUM, with its greatest value reflected in improving long-term survival (1-year, 2-year, and 3-year OS) — a finding consistent with its FDA approval basis. While ORR and DCR provide supplementary evidence of therapeutic benefit, radiological response rates (e.g., CR, PR) are limited in fully capturing its clinical value. Safety concerns include high CRS incidence (mostly low-grade and manageable) and variable grade ≥3 TRAE rates. No previous treatment received patients may derive greater benefits. Limitations (heterogeneity, HLA-A*02:01 restriction, limited long-term data) highlight the need for more high-quality studies to validate long-term efficacy/safety, expand applicability to broader populations, and explore combination therapies. Additionally, circulating tumor DNA (ctDNA) may serve as a more sensitive efficacy biomarker than radiological responses, warranting further investigation.

## Data Availability

The original contributions presented in the study are included in the article/**Supplementary Material**. Further inquiries can be directed to the corresponding author.

## Supplementary material

Supplementary Figure 1 Sensitivity analysis: CRS.

Supplementary Figure 2 Subgroup analysis: CR.

Supplementary Figure 3 Subgroup analysis: PR.

Supplementary Figure 4 Subgroup analysis: SD.

Supplementary Figure 5 Subgroup analysis: ORR.

Supplementary Figure 6 Subgroup analysis: DCR.

Supplementary Figure 7 Subgroup analysis: One-year OS.

Supplementary Figure 8 Subgroup analysis: Two-years OS.

Supplementary Figure 9 Subgroup analysis: ≥3 TRAEs.

Supplementary Figure 10 Subgroup analysis: CRS.
