# Supplementary material for: Evaluating the efficacy and safety of tebentafusp in the treatment of metastatic uveal melanoma: a 2025 update systematic review and meta-analysis
Source: Front Oncol. 2025 Oct 15;15:1667282. doi: 10.3389/fonc.2025.1667282 (PMC12568024; doi:10.3389/fonc.2025.1667282)
Supplement: Supplementary file 11 [file Table1.docx]

**Supplementary Table 1** Assessment of non-randomized controlled trials in the version of MINORS.

| Study | A clearly  stated  aim | Inclusion of  consecutive  patients | Prospective  collection of  data | Endpoint  appropriate  to the study  aim | Unbiased  assessment  of endpoints | Follow-up  period  appropriate  to the major  endpoint | Loss to  follow  up not  exceeding  5% | Prospective  calculation  of the study  size | Total score |
| --- | --- | --- | --- | --- | --- | --- | --- | --- | --- |
| Shoushtari A.N2021 | 2 | 2 | 2 | 1 | 2 | 0 | 2 | 1 | 12 |
| Mark R. Middleton2022 | 2 | 2 | 2 | 1 | 2 | 1 | 2 | 2 | 14 |
| Richard D. Carvajal2022 | 2 | 2 | 2 | 1 | 2 | 2 | 2 | 2 | 15 |
| Takami Sato2022 | 2 | 2 | 2 | 1 | 2 | 2 | 2 | 2 | 15 |
| Natalia M. Roshardt Prieto2023 | 2 | 2 | 2 | 1 | 2 | 1 | 2 | 2 | 14 |
| Jessica C. Hassel2023 | 2 | 2 | 2 | 2 | 2 | 2 | 2 | 2 | 16 |
| Dirk Tomsitz2023 | 2 | 2 | 2 | 1 | 2 | 1 | 2 | 1 | 13 |
| Andrisha Jade Inderjeeth2023 | 2 | 2 | 2 | 1 | 2 | 0 | 2 | 1 | 12 |
| Mailly-Giacchetti, L2023 | 2 | 2 | 2 | 1 | 2 | 2 | 2 | 1 | 14 |
| Ribeiro, M. F2023 | 2 | 2 | 2 | 0 | 2 | 2 | 2 | 1 | 13 |
| Alexander Maurer2024 | 2 | 2 | 2 | 1 | 2 | 1 | 2 | 2 | 14 |
| Manuel Rodrigues2024 | 2 | 2 | 2 | 1 | 2 | 1 | 2 | 1 | 13 |
| Joseph J Sacco2024 | 2 | 2 | 2 | 1 | 2 | 2 | 2 | 2 | 15 |
| Lucille VITEK2024 | 2 | 2 | 2 | 2 | 2 | 2 | 2 | 2 | 16 |
| Gradone, A 2024 | 2 | 2 | 2 | 0 | 2 | 0 | 2 | 1 | 11 |
| Nathan, P2024 | 2 | 2 | 2 | 2 | 2 | 1 | 2 | 1 | 14 |
| Piccin, L2024 | 2 | 2 | 2 | 1 | 2 | 0 | 2 | 1 | 12 |
| J M Piulats 2024 | 2 | 2 | 2 | 0 | 2 | 1 | 2 | 1 | 12 |
